# South African Consumers’ Knowledge, Opinions and Awareness of Whole Grains and Their Health Benefits: A Cross-Sectional Online Survey

**DOI:** 10.3390/nu15163522

**Published:** 2023-08-10

**Authors:** John R. N. Taylor, Colin D. Rehm, Henriëtte L. de Kock, Suné Donoghue, Andrew Johnson, Chanelle Thompson, Yulia Berezhnaya

**Affiliations:** 1 Department of Consumer and Food Sciences, University of Pretoria, Pretoria 0002, South Africa; riette.dekock@up.ac.za (H.L.d.K.); sune.donoghue@up.ac.za (S.D.); 2 PepsiCo Global Research & Development, Life Sciences, PepsiCo, Purchase, NY 10577, USA; colin.rehm@pepsico.com; 3 Beyond Insights, Durban North 4051, South Africa; andrew@beyondinsights.co.za; 4 PepsiCo South Africa, Consumer Insights, PepsiCo, Cape Town 7530, South Africa; chanelle.thompson@pepsico.com; 5 PepsiCo Global Research & Development, Life Sciences, PepsiCo, Cape Town 7530, South Africa; yulia.berezhnaya@pepsico.com

**Keywords:** whole grains, health benefits, consumers, South Africa, socio-demographics, objective knowledge, subjective knowledge, developing countries

## Abstract

Evidence indicates that whole-grain food consumption reduces the risk of cardiovascular disease, type-2 diabetes, and some cancers. Increasing whole-grain consumption in developing countries is likely to significantly benefit the health of the population. However, there is very limited information on consumer whole-grain knowledge, attitudes, and behaviors in developing countries. An online cross-sectional survey was conducted among 1000 South African consumers with sufficient income to make food purchase choices and who were generally representative in terms of gender, age, and ethnicity. Most respondents (64%) were confident of their whole-grain knowledge. However, 60% of all participants selected incorrect definitions of whole grains. Whilst most correctly identified common cereals as whole grains, at most 50% of participants correctly identified common whole-grain foods. Also, whilst most (67%) thought that they were consuming enough whole grains, the majority (62%) underestimated the recommended level of consumption. Furthermore, respondent knowledge regarding whole-grain food attributes and the health benefits of whole-grain consumption was generally poor. Clearly, consumer-focused strategies are needed in developing countries to increase whole-grain food consumption to help the broader population achieve a healthy and sustainable diet. Actions proposed include: simple-to-understand information on whole-grain content relative to recommendations on food product labels, the provision of whole-grain foods in school nutrition schemes, and coordinated social and behavior change communication initiatives.

## 1. Introduction

While definitions vary slightly by country and across research studies, generally, whole grains are defined as products that contain all three anatomical components of cereal grains or pseudocereal grains, i.e., the endosperm, germ, and bran [1,2]. This may include products that are unprocessed (except for removing the inedible hull/husk), such as brown rice or quinoa, and products that have undergone some processing, such as breakfast cereals made from whole-grain ingredients or bread made from whole-wheat flour. Whole grains are typically higher in many micronutrients than their refined grain counterparts and are specifically higher in insoluble dietary fiber [3]. Epidemiologic studies suggest that consumption of whole-grain foods is associated with a reduced risk of diabetes, cardiovascular disease, and some cancers [4,5,6,7].

The Global Burden of Disease (GBD) project estimates that sub-optimal whole-grain food consumption (defined as <140–160 g/d, a level which is equivalent to five servings, e.g., five slices of whole-grain bread), is a leading dietary risk factor causing sub-optimal health. This issue ranks just below sodium globally, but is ahead of sub-optimal consumption of legumes, fruits, vegetables, and sugar-sweetened beverages [8]. As such, increasing whole-grain consumption at the population level is an important public health goal and many countries’ food-based dietary guidelines recommend that individuals increase their whole-grain intake.

However, there are relatively sparse data on the actual consumption of whole grains at the population level. For South Africa, a large middle-income country, there are no published data, making it challenging to understand whole-grain trends and patterns of consumption. Part of this challenge is due to limited dietary surveillance surveys and the fact that many dietary surveys are not designed to quantify whole-grain consumption due to the absence of whole-grain definitions in the underlying databases (e.g., the National Dietary and Nutrition Survey [NDNS] in the United Kingdom) [1,9]. While studies of consumption patterns are important, understanding consumers’ attitudes and their subjective (i.e., what consumers perceive they know, self-assessed) and objective knowledge of whole grains (i.e., what consumers actually know based on food composition and regulation) is equally critical. A limited number of such studies have been conducted in Australia [10,11,12], Malaysia [13,14], Singapore [15], the UK [16], and the US [17]. These studies generally observe that knowledge of whole grains is low and that consumers are generally unaware of the health benefits of whole grains. Most of these data come from small, non-representative samples in high-income contexts; little is known about consumer understanding and the perceptions of whole grains in middle-income countries. Lacking these data can hinder the development of population-based interventions and campaigns that seek to increase consumer knowledge of whole grains and to increase whole-grain consumption. Furthermore, these data can support efforts by food manufacturers that produce and market whole-grain foods.

The purpose of this study was to obtain quantitative insights as to what consumers in a large middle-income developing country (South Africa) know and think about whole grains. This research was conducted with the aim of informing strategies to enable whole grains to become core components of healthy and sustainable diets in such countries. To this end, we collected data on knowledge, attitudes and behaviors around whole grains and whole-grain foods from 1000 South African consumers. The primary objectives of the present report are to: (1) to describe patterns in consumer perceptions of and knowledge about whole grains for the total sample and by socio-demographic characteristics (e.g., age, gender, and socioeconomic status); and (2) to evaluate the association between subjective and objective knowledge regarding whole grains. Finally, taking the findings from both objectives into account, we aim to propose initiatives that are suitable for implementation in developing countries by government health departments, the food industry, professional societies, and other organizations in order to support the goal of substantially increasing the consumption of whole-grain foods.

## 2. Materials and Methods

### 2.1. Study Design and Implementation

A cross-sectional quantitative online survey of 1000 adults aged 18–64 years (y) residing in all areas of South Africa, but primarily in the Gauteng, KwaZulu-Natal and Western Cape Provinces, was conducted in December 2022.

The questionnaire comprised a total of 26 questions concerning three different but interrelated topics: respondents’ breakfast consumption habits and attitudes towards breakfast; an exploration of their whole-grain knowledge, opinions and health benefits awareness; and their self-reported health and attitudes regarding health and wellness. The questions were structured and closed-ended, using multiple-choice questions, single-selection and multi-select answer options, ranking scale questions, and Likert-type rating scale questions. Specifically, they used a 9-point scale ranging from strongly disagree to agree strongly, a 5-point scale ranging from poor to excellent, a 5-point scale ranging from not at all concerned to very concerned.

The questions concerning whole grains were in part modeled on those used in published survey research concerning consumers’ objective whole-grain knowledge and attitudes to whole grains, notably those found in the work of Foster et al. and McMackin et al. [12,16]. The five options that measured respondents’ subjective knowledge of whole grains were based on Flynn and Goldsmith’s standardized scale, which measures subjective knowledge [18]. The lists of attributes of whole-grain products and health benefits of whole-grain food consumption were derived from an internal qualitative market research survey. The questions were written to take into account particular foods consumed in South Africa, as well as the current and proposed South African whole-grain food labeling legislation. Furthermore, they were phrased using terms that South African consumers would understand [19,20]. Validation of the instrument involved refining the draft questionnaire by means of two consumer focus discussion groups, each comprising eight people representative of adult South Africans. Following this, the wording of the questions was reviewed and amended by members of the panel provider and the authors. As an example of the changes made, Question 24, which gave options for the recommended daily consumption of whole grains, was changed from asking about food weights to servings of typical whole-grain foods (see S1 Whole grains survey questions). The questions sought to obtain the following information:

- How well respondents thought they understood the term whole grains (subjective knowledge) and how well they understood the term (objective knowledge).
- Their objective knowledge as to which food ingredients are whole grains and which food products generally contain whole grains.
- What attributes, including sensory characteristics, cost and affordability, product labeling, product availability, and consumer marketing, they most associated with whole-grain foods.
- What they understood to be the major health benefits of consuming whole grains.
- Whether they knew the quantity of whole grains it is recommended that people consume and whether they thought that they were consuming enough whole grains.

The actual question wording and response options relevant to the present research are provided in Supplemental Materials.

Participation in the study was voluntary, performed with full informed consent, and anonymous. Participants could also opt out at any time. Respondents received a ZAR 30 store voucher as an incentive for participating. Ethical approval for the study was provided by the University of Pretoria’s Ethics Committee (ethics number NAS357/2022).

Participants were recruited by Borderless Access (a market research company) via their database of some 300,000 South Africans. Prior to fielding, the questionnaire was pre-tested internally in order to identify ambiguous wording and online questionnaire design problems that could result in misinterpretation. An e-mail was sent out to invite people in the database to participate in the study who fitted the criteria. Respondents provided informed consent by clicking on a link after reviewing the study synopsis, research procedure and their rights as participants. Those responding were pre-screened to ensure quotas for gender, population group, age and living standard). Briefly, living standard was assessed in accordance with the South African Living Standards Measure (LSM), a local measure of socioeconomic status that functions regardless of ethnicity, income or education, based on the ownership of certain durable goods (e.g., appliances, cell phone, air conditioner, etc.) and access to specific service (e.g., tap water, flush toilet, etc.) [21]. Persons in LSM deciles 5–10 can be considered as those with sufficient disposable income to make choices as to what food products to purchase and are likely to consume more diverse diets than individuals in the lower-LSM deciles (i.e., 1–4). Data were obtained over a period of 10 days from equal numbers of females and males in approximate proportion to South Africa’s population groups.

### 2.2. Statistical Analysis

To describe the association between socio-demographic factors and whole-grain knowledge, attitudes, and behaviors, heatmaps) were constructed that use color intensity to visually demonstrate percentage values for the ease of detecting patterns and within-group differences. Chi-squared tests were used to assess differences in the proportion of respondents answering each question by age, gender, LSM level, and self-reported health status.

The responses to how well respondents thought they understood the term whole grains (subjective knowledge) were grouped into two categories: respondents who believed they were more knowledgeable about whole grains “Among my circle of friends, I am the expert on whole grains”; “I know what whole grains mean”; and those who were less knowledgeable “I do not feel very knowledgeable about whole grains”; “When it comes to whole grains, I really do not know a lot”; “Compared to most other people, I know less about whole grains”. The objective knowledge score per respondent was calculated based on 1. the correct answer for the term whole grains (maximum 1 mark/point), 2. the number of correct answers chosen and incorrect answers not chosen concerning whole-grain items (ingredients) (maximum 20 marks), and 3. the products that generally contain whole grains (maximum 26 marks). The total objective knowledge score comprised 47 marks (100% correct answers). The relationship between knowledge (subjective and objective) and grouping characteristics was assessed using the Kruskal–Wallis test. Multiple pairwise comparisons were performed using Dunn’s procedure or a Chi-squared test.

Analyses were performed using XLstat (Addinsoft, Paris, France) and Stata 17.0 (College Station, TX, USA). An alpha-level of 0.05 was used to determine statistical significance. As a descriptive study providing data on whole-grain knowledge, attitudes and behaviors for the first time, there was no correction for multiple comparisons.

## 3. Results

### 3.1. Participant Characteristics

Participant characteristics are provided in Table 1 and are generally representative of South Africans with an LSM value of ≥5. Sixty percent of respondents were in the LSM deciles 5–7, i.e., those with intermediate access to wealth, and forty percent were in LSM deciles 8–10, i.e., those with the highest access to wealth. These LSM groups together represent approximately 40% of the South African population. There was an equal split by gender, and most were between 25–44 y. About ¾ of subjects identified as Black, with the remaining identified as Coloured/Indian (13%) or White (10.6%). The Coloured and Indian groups were considered together in order to obtain a meaningful sample size. Sixty percent of the sample had an LSM between 5 and 7. A plurality of participants resided in Gauteng. In terms of general health status, about 19% of participants had excellent self-reported health and 29% reported very good health. Only about 13% of subjects reported having fair or poor health.

### 3.2. Univariate Results and Socio-Demographic Factors Associated with Whole Grains Knowledge, Attitudes and Behaviors

#### 3.2.1. Whole-Grain Knowledge

**Figure 1:**
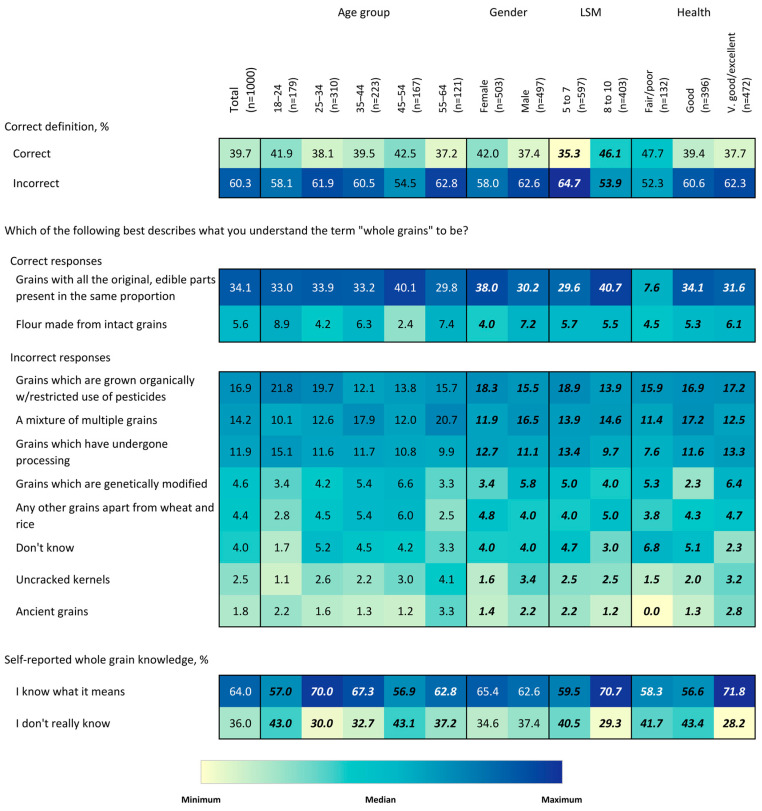
Heatmap of providing correct definition to whole grains, specific definition provided, and self-reported whole grain knowledge, overall and by age group, gender, LSM level and self-rated health status. Values in bold/italics are statistically significant at the 0.05 level. Colors correspond to size of values corresponding to colors in the legend. Colors are coded for each question separately.

Regarding respondents’ subjective knowledge about whole grains, 64% of respondents were confident that they knew what whole grains are, whereas 36% stated that they knew nothing or very little about whole grains (Figure 1, Table 2). There was no significant effect of gender (*p* > 0.05). However, a substantially higher proportion (*p* < 0.05) of the higher-LSM group claimed to know what whole grains are. In agreement with this, a significantly higher percentage of the lower-LSM group responded that they know nothing or very little about whole grains. Substantially more respondents in the age groups 25–34 y claimed to know what whole grains mean, some >55% of respondents. Some differences in self-reported whole-grain knowledge were also observed by health status. Those with very good/excellent health were more likely to report being an expert among their peers, whereas individuals with fair/poor health were more likely to report that they “don’t really know” the definition.

Concerning objective knowledge as to what whole grains actually are, only 34% of respondents selected the description, “grains with all the original, edible parts present in the same proportion as when the grain was growing in the fields” (Figure 1, Table 2), which is based on the Whole Grain Initiative consensus global definition of whole grain as a food ingredient [22]. Another 6% selected “flour made from intact kernels”, a definition that is in accordance with the proposed South African legislation definition of “wholegrain flour/meal”, i.e., “wholegrain flour/meal” means flour obtained by the milling of dehulled or dehusked intact whole grains which, after milling, still contains all the components [20]. Both definitions can be considered correct. The most common misconceptions were that whole grains are the same as organic foods (17%) and that they are multi-grains (i.e., mixtures of grains) (14%). A higher proportion (*p* < 0.05) of the higher-LSM group selected the accepted definitions (42% vs. 35% for the lower-LSM group), whereas a significantly higher proportion of the lower-LSM group selected the organic grain description (19% vs. 14% for the higher-LSM group). A considerably, but not statistically significantly, higher proportion of respondents in the 44–55 y group selected the accepted definition (40%) than those in the other age groups (highest 34%). Also, a significantly higher proportion of women selected the accepted definition (38% vs. 30% for men). However, significantly more males selected the “flour from intact grains” description (7%) than females (4%), and more men incorrectly selected the option, “a mixture of multi-grains”. Specific definitions varied by self-reported health status. The percentage providing the correct definition of whole grains did not differ significantly by self-reported health status.

**Figure 2:**
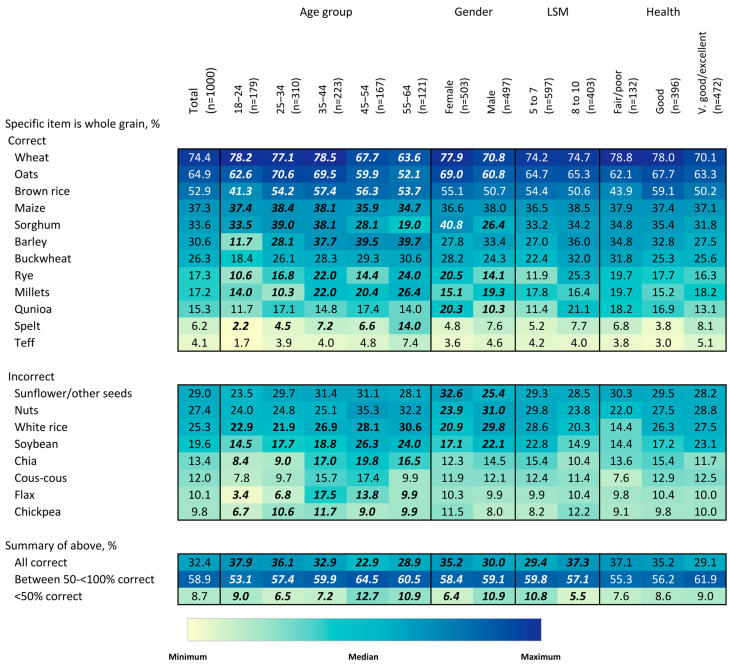
Heatmap describing the percentage of respondents identifying each item to be a whole grain or not, overall and by age group, gender, LSM level and self-rated health status. Values in bold/italics are statistically significant at the 0.05 level. Colors correspond to size of values corresponding to colors in the legend. Colors are coded for each question separately.

To determine whether respondents knew which food items, alternatively described as ingredients [22], were whole grains, they were asked to select from a list of various grain-type items. The top six selections made by the respondents were correct and, in descending order, were wheat, oats, brown rice, maize, sorghum, and barley (Figure 2). Wheat, oats, and brown rice were correctly selected by more than 50% of the respondents, with 74% of respondents identifying wheat as a whole grain. The two pseudocereals listed, buckwheat and quinoa, were only correctly identified as whole grains by a minority of respondents, 26% and 15%, respectively. Older adults were less likely to identify actual whole grains, e.g., wheat, oats, and sorghum, e.g., 19.0% of 55–64 y vs. 33.5% for 18–24 y for sorghum. A significantly higher proportion of women than men (35% vs. 30%) selected all options correctly (*p* < 0.05). There was no significant influence of LSM group (socioeconomic status) on the correctness of selections. The most common misconceptions were that seeds (29% of respondents), nuts (27%), white rice (25%) and soybeans (20%) were whole grains. Further, more subjects incorrectly identified chickpea, flax, chia, and soybean as whole grains, which are pulses and oilseeds, than spelt and teff, which are cereal grains. Individuals reporting fair/poor health were more likely to report that wheat is a whole grain but less likely to report brown rice as such. For items which were not whole grains, individuals with very good/excellent health were more likely to incorrectly report white rice and soybean as whole grains.

**Figure 3:**
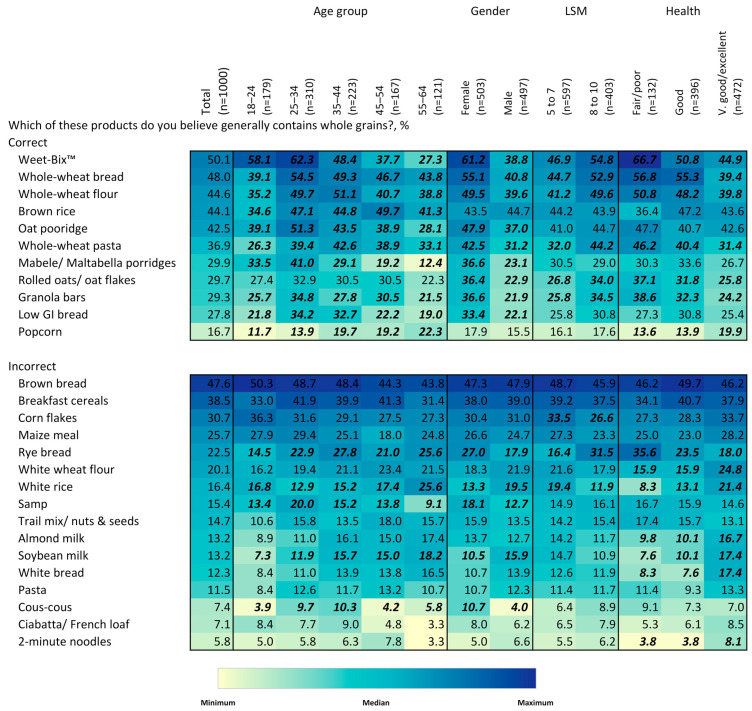
Heatmap of percentage identifying specific products as generally containing whole grains, overall and by age group, gender, LSM level and self-rated health status. Values in bold/italics are statistically significant at the 0.05 level. Colors correspond to size of values corresponding to colors in the legend. Colors are coded for each question separately.

The highest percentage of respondents selecting a particular food product as generally containing whole grains was only 50%, which was for Weet-Bix^TM^ (Pioneer Foods Groceries (PTY) Ltd; Tyger Valley, South Africa), a flaked whole-wheat ready-to-eat breakfast cereal brand consumed in the form of a biscuit (Figure 3). The next highest percentage was for whole-wheat bread at 48%. By far the most common misconception was that brown bread generally contains whole grains, also believed by 48% of respondents. This was followed by breakfast cereals (39%) and corn flakes (31%). With the exceptions of brown rice and popcorn, a significantly higher proportion of women correctly selected the products containing whole grains compared to men. Generally, a significantly lower proportion of the youngest age group, 18–24 y, and of the oldest age group, 55–64 y, correctly selected the products containing whole grains. Except for brown rice, a significantly higher proportion of the higher-LSM group correctly selected the products containing whole grains. Individuals reporting fair/poor self-rated health were more likely to report Weet-Bix^TM^, whole-wheat bread, whole-wheat flour, and whole-wheat pasta as being whole grains than individuals with very good/excellent health. As with the prior results for specific ingredients, individuals with very good/excellent health were more likely to misidentify white rice, almond milk, soybean milk and white bread as containing whole grains.

#### 3.2.2. Objective versus Subjective Whole-Grain Knowledge

There was an association between subjective knowledge about whole grains and objective knowledge as to what whole grains are (Table 2). Those with higher levels of subjective knowledge displayed higher objective knowledge about whole-grain ingredients, but not products containing whole grains, compared to those with low subjective knowledge. Females and higher-LSM respondents had greater objective knowledge about whole grains compared to males (score of 29.1 vs. 27.4 for men) and lower-LSM respondents (score of 29.2 for high LSM group vs. 27.7 low LSM group). A much higher proportion of higher-LSM respondents considered themselves to have good subjective knowledge compared to lower-LSM respondents (60% vs. 40%). The objective knowledge of young consumers (18–24 y) and older consumers (45–64 y) about whole grains was worse than that of those between 25 and 44 years of age. The same trend was evident for subjective knowledge.

#### 3.2.3. Whole-Grain Attitudes and Behavior

**Figure 4:**
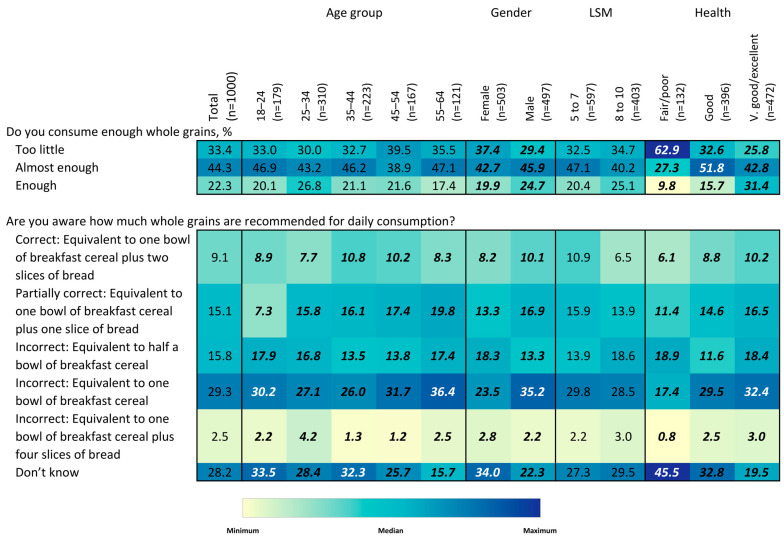
Heatmap of reported whole-grain consumption and awareness of recommended amounts to-be consumed, overall and by age group, gender, LSM level and self-rated health status. Values in bold/italics are statistically significant at the 0.05 level. Colors correspond to size of values corresponding to colors in the legend. Colors are coded for each question separately.

Overall, 22.3% of respondents reported that they consumed enough whole grains, 44.3% said they consumed almost enough, and 33.4% indicated they consumed too little (Figure 4). There were no significant differences in responses to this question by age group or LSM level. Women were more likely to report that they consumed too little (37.4% vs. 29.4% for men), while men were more likely to report consuming enough (24.7% vs. 19.9% for women). By self-reported health status, only 9.8% of individuals with fair/poor health reported consuming, enough compared to 31.4% of those with very good/excellent health (*p* < 0.05). While few South African adults reported consuming enough whole grains, knowledge regarding the level of whole grain consumption recommended was quite low. Specifically, only 9.1% of respondents indicated that the recommended amount was one bowl of breakfast cereal and two slices of bread, which approximately represented the recommended amount. More than a ¼ of respondents indicated that they did not know the recommended amount. Older adults were less likely than younger adults to report that they did not know the recommended amount (15.7% vs. 25.7% or higher), as were men (22.3% vs. 34% for women) and individuals with very good/excellent self-reported health (19.5% vs. 45.5% for those reporting fair/poor health). No differences were observed by LSM level.

**Figure 5:**
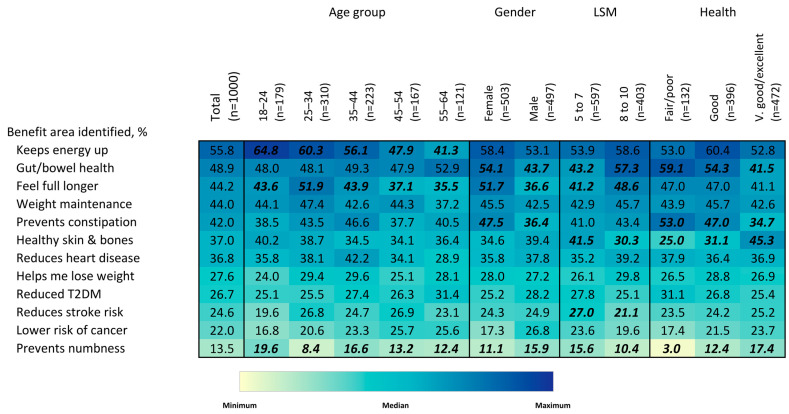
Heatmap of reported benefits of whole grains, overall and by age group, gender, LSM level and self-rated health status. Values in bold/italics are statistically significant at the 0.05 level. Colors correspond to size of values corresponding to colors in the legend. Colors are coded for each question separately.

Participants were asked to describe perceived benefits of whole grains using a list of 12 options (Figure 5). Overall, the number one benefit described was “keeps energy levels up” (55.8%), followed by “gut/bowel health” (48.9%), “feeling full longer” (44.2%), “weight maintenance” (44.0%), and “prevents constipation” (42.0%). Benefits related to clinical outcomes were less frequently reported, with “reduces heart disease” (36.8%) being the most reported of these options. There was a strong relationship between age and some of the benefits identified, with younger adults being more likely to report that whole grains “keep energy levels up” and make you “feel full longer” than older adults, (64.8% vs. 41.3%) and (43.6% vs. 35.5%), respectively, for adults 18–24 y vs. adults 55–64 y. Women were more likely to report a benefit related to “gut/bowel health”, “feeling full longer” and “prevents constipation” (*p* < 0.05 for each). The only benefit area men were more likely to report was “prevents numbness”. Individuals in the higher-LSM group were more likely to report a benefit for “gut/bowel health” and “feeling full longer”, while the lower-LSM group was more likely to report benefits for “healthy skin & bones” and “prevents numbness”. Participants with fair/poor self-reported health were more likely to report benefits related to “gut/bowel health” and “prevents constipation”, while individuals that self-reported very good/excellent health were more likely to report benefits related to “healthy skin & bones” and “prevents numbness”.

#### 3.2.4. Attributes of Whole-Grain Food Products

**Figure 6:**
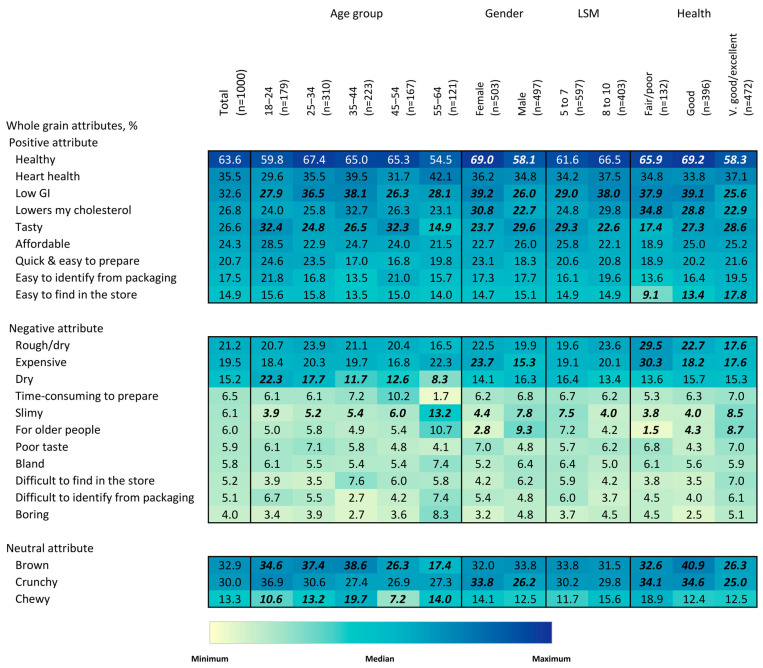
Heatmap of reported attributes of whole-grain products, overall and by age group, gender, LSM level and self-rated health status. Values in bold/italics are statistically significant at the 0.05 level. Colors correspond to size of values corresponding to colors in the legend. Colors are coded for each question separately.

Concerning consumer associations with the attributes of whole-grain food products, the attribute that most consumers associated with whole-grain products was “healthy”, followed by “heart health”, “brown”,” low GI” and “crunchy” (Figure 6). Variations in the order of selection by percentage were evident when comparing by gender, age and LSM groupings. It was also noted that young consumers (18–25 y), in comparison to the other age groups, associated the sensory properties of “brown”, “crunchy” and “tasty” with these products more often than their specific health-related attributes. More than 40% of respondents in the 55–64 y group associated “heart health” with whole-grain products, which was more than any other age group. Women associated “low GI” and “lowers my cholesterol” with whole-grain products more often than men, 30.8% vs. 22.7%. Higher proportions of the lower-LSM respondents associated the attributes “affordable” and “tasty” with whole-grain products than higher-LSM respondents and did so more often. In contrast, percentagewise, greater numbers of the higher-LSM respondents associated health-related attributes with whole-grain products than the lower-LSM respondents.

## 4. Discussion

### 4.1. Whole-Grain Knowledge

Concerning South African consumers’ subjective knowledge about whole grains, 64% of respondents were confident they knew what whole grains are, indicating a reasonable level of subjective knowledge. When considering consumers’ subjective knowledge about other foods, this is higher than, for example, South African consumers’ subjective knowledge about Karoo lamb [23], but similar to Uruguayan consumers’ knowledge about olive oil [24]. Consumers’ subjective knowledge about whole grains has not previously been systematically evaluated. A small study in the UK (n = 43) indicated a lack of understanding around whole-grain foods [10] and a larger study in Australia (n = 448) revealed that 96% of consumers had previously heard of whole grains and whole-grain foods [12].

Regarding consumers’ objective knowledge of what whole grains are, the proportion of respondents selecting the two correct definitions of whole grains (40%) was lower than found in the study carried out in Australia (49%) [12]. This is probably related to gender and socioeconomic status differences between the two studies. In the Australian study, 87% of the respondents were females and 72% held post-school qualifications. As indicated, in the South African study, a higher proportion of females and respondents in the higher-LSM group selected the accepted definition of whole grains. Similarly, a study in Croatia (a middle-income country) revealed that women and those with a university education had a greater general knowledge about dietary fiber with specific regard to whole-grain foods [25].

Concerning South African consumer knowledge as to which food items, alternatively referred to as ingredients [22], are whole grains, the proportion of correct answers for the common grains was very similar to that of the Australian study; oats 65% (this study) vs. 56% (Australian study), brown rice 53% vs. 56%, and white rice 25% vs. 27% [12]. However, for the less common grains, the proportion of correct answers from South African consumers was far lower; rye 17% vs. 55%, quinoa 15% vs. 58%, and teff 4% vs. 25%. This is probably related to lower exposure to “super food”-type food trends and the lower spending power of South African consumers. It is significant that a comparable and substantial proportion of consumers in both Australia and South Africa thought that white rice is a whole grain. Further, a comparable proportion of South African consumers thought that seeds, nuts, and soybean are whole grains. An earlier small consumer focus group study (n = 43) in the UK revealed a similar type of confusion as to whether grains such as lentils were whole grains [16]. Clearly, the “grain-type” form of these foodstuffs is a common factor that results in the misconception. This has important implications for consumer education about whole grains.

Regarding South African consumers’ knowledge about which food products generally contain whole grains, as indicated, there were three notable misconceptions: brown bread, breakfast cereals, corn flakes. The brown bread misconception is easy to understand, not only in terms of being “brown” in the name. In South Africa, basic brown bread, the most common brown bread product, is made from white bread flour plus a portion of bran, minus the germ, meaning that approximately 87% of the wheat kernel by weight is included in the flour and 13%, essentially the germ and the fine bran, is not included [26]. The germ is removed to increase the storage life of the flour. The situation is made more complex because there are also premium brown breads that contain intact wheat kernels. The breakfast cereals misconception is probably related to the fact that, although there are numerous whole-grain breakfast cereal brands available in South Africa, the majority of breakfast cereals, such as corn flakes and most extruded-type breakfast cereals, do not contain whole grains. Further, there is the issue that respondents generally understood that whole grains are cereals and that breakfast cereals are commonly simply referred to as “cereals”. Concerning the corn flakes misconception, corn flakes are produced from flaking grits, which are essentially the intact maize endosperm, minus the bran and germ [27]. Flaking grits are produced via the process of “degermination” during maize milling. This misconception is probably related to the fact that corn flakes are widely described, for example in Wikipedia, as “made from toasting flakes of corn (maize)”. This means that, by analogy, it may be assumed that they are whole grains as with oat flakes [28]. Some brands also claim that their products are sources of fiber, which may contribute to some consumers identifying them as whole-grain products. While rye bread in some countries may be considered a whole-grain product, the vast majority of rye breads available in South Africa are either made from rye flours of less than 100% extraction rate, or from a blend of these rye flours and white bread flour, i.e., so-called “soft rye” bread.

It is more difficult to make direct comparisons with the results of the Australian study concerning products containing whole grains than it is for grain food items (ingredients) [12]. The wording of the questions posed in the survey questionnaires were somewhat different, different names are used for some food products, and the same name may be used for products with different compositions. Nevertheless, some useful comparisons can be made for correct answers; Weet-Bix^TM^ 50% (present study) vs. 60% (Australian study), whole-wheat pasta 37% vs. 58%, and whole-wheat flour 20% vs. 8%, and also for incorrect answers; white bread 15% vs. 25%, and white flour 25% vs. 27%. Overall, it appears that the level of knowledge as to whether specific food products contain whole grains was similar across the South African and Australian consumer groups. As the proportion of correct answers was at most 60%, this finding, like that concerning grain ingredients, indicates a strong need for consumer education concerning whole grains.

Importantly, in the present study, there were some clear trends regarding respondent age, socioeconomic status, and gender across the three questions probing objective knowledge about whole grains. The oldest age group, 55–64 y, and the lower-LSM group consistently gave the lowest percentage of correct answers. This may have significant health implications as, for example, the incidence of constipation seems to be higher in older people and those of lower socio-economic level and changes in the gut microbiome with aging may be involved in the increased incidence of inflammatory conditions [29,30]. Further, in many countries individuals of lower socioeconomic status typically consume fewer whole grains, which may contribute to health disparities [9,31,32]. On the other hand, in many of the same populations, older individuals consume more whole grains than younger adults, presenting somewhat of a paradox between objective knowledge and actual consumer behavior [9,31]. When assessing by gender, it was found that a consistently higher proportion of females provided the correct answers, which may reflect the fact that women are generally found to be more interested in healthy eating than men [33]. Women are also more likely than men to be responsible for grocery shopping and act as household gatekeepers of food purchases [34].

### 4.2. Attributes of Whole-Grain Food Products

The perceived attributes of all food products are dependent on consumer expectations and quality perceptions, which are based on intrinsic and extrinsic product attributes. Intrinsic attributes, i.e., its sensory attributes and nutrient composition, are inherent to the product [35,36]. In contrast, extrinsic attributes are related to the product but are not physically part of it. Examples include price, brand name, product packaging, labeling, nutritional and health claims, product promotion, and availability [36,37].

Clearly, the respondents strongly associated whole-grain products with having health-promoting attributes, a consequence of both intrinsic and extrinsic factors. In fact, healthy was the only attribute selected by most respondents (63.4%), and all four health-related attributes ranked among the top six attributes selected. It is, however, difficult to attribute a reason as to why higher proportions of respondents who considered that their health was only poor/fair or good selected the health-promoting attributes of whole-grain foods compared to those who considered their health to be very good/excellent. There is a paucity of comparative data in this area. A focus group study in Australia indicated that participants who consumed some whole grains recognized that whole-grain foods were a healthier option than refined-grain foods [11]. A qualitative study in Northern Ireland similarly observed that health-related factors are important facilitators of whole-grain consumption [16]. In contrast to the older consumers, young consumers identified the sensory properties of whole grains more often than the specific health-related attributes. This may be due to older consumers generally being more at risk of suffering from diet-related diseases and health problems.

Considering the intrinsic sensory attributes, these were dominated by ‘brown’ (Figure 6), a characteristic of all whole-grain foods that emerges as a result of the anthocyanin pigments in the bran and ‘crunchy’, a characteristic typical of products such as branny and granola-type breakfast cereals and granola bars [38,39,40]. A focus group study of young Singaporean adults (n = 30) indicated that the “dull” brown color of most whole-grain products may discourage individuals from their consumption [15]. Whether this is universal is not known. For this reason, in this present study, brown and crunchy are categorized by the authors as neutral attributes (but not necessarily the respondents).

‘Tasty’ was the only other sensory attribute selected by >25% of respondents. Since ‘poor taste’ and ‘bland’, essentially the opposite sensory attributes, were selected by <6% of respondents, this suggests that this group of South Africans, those who likely have sufficient disposable income to make choices as to what food products to purchase, viewed the sensory attributes of whole-grain foods positively. This finding is somewhat similar to that of the Australian focus group study, where participants who consumed some whole grains stated that they preferred their taste to that of the refined-grain counterparts [11]. Because taste is well known to be a primary driver of food choice, these results are encouraging, but likely require validation in other populations and larger studies [41].

The fact that more respondents selected the positive extrinsic food product attributes of easy-to-find and -identify, affordable and quick- and easy-to-prepare than their opposites could, at first sight, suggests that obtaining, purchasing, and preparing whole-grain foods are not great barriers to their consumption in developing countries like South Africa. However, it would be unwise to draw such a conclusion. Firstly, the fact that >30% of respondents incorrectly identified products such as brown bread, breakfast cereals, and corn flakes as generally containing whole grains casts some doubt on the validity of the data. Moreover, as stated, this group of consumers only represent approximately 40% of the South African population. Further, even among this more affluent group, there was a relatively small difference between those who selected whole-grain products as being affordable, 24.3% of respondents, versus those who selected them as expensive, 19.5%. Also, a survey of adults in Malaysia [14], also a developing country, revealed that the perceived high cost of whole-grain foods, the problem of identifying them, lack of knowledge concerning their preparation, and inferior sensory characteristics were all predominant barriers to their consumption.

### 4.3. Approaches to Increasing Whole-Grain Consumption-Specific Considerations for Developing Countries

A key question arising from these research findings is what needs to be done in developing countries to substantially increase the consumption of whole-grain foods in order to support the goal of a more healthy and sustainable diet for the broader population. The consumption of whole grains by a population is dependent on several factors, including consumer awareness of the health benefits, recommended amounts for consumption per day, accessibility of products containing whole grains in retail stores, visibility of these products through effective labeling and communication, sensory properties of those products, and affordability. It is also important to increase whole-grain intake without increasing the intake of sodium, added sugars, saturated fats, or other dietary constituents whose intake should be limited.

One commonly employed first step to help improve consumer awareness is the incorporation of whole-grain recommendations into dietary guidelines [42]. Specific implementations vary globally, but there is some evidence suggesting that these recommendations lead to the increased consumption of whole grains. In the US, whole grains first appeared prominently in dietary guidelines in 2005. Data from NHANES (US) indicate a notable increase in whole-grain consumption across all age groups from 2003/2004 to 2013/2014. During this period, whole-grain intake rose from 0.6 ounce-equivalents per day to 0.9. Simultaneously, the consumption of refined grains significantly decreased, moving from 6.3 to 5.7 ounce-equivalents per day, an approximate 10% decrease [43]. A recent USDA study further revealed that the increase in whole-grain consumption among children was noteworthy, particularly from school meals [44], suggesting that school food policy, an important downstream consequence of the inclusion of whole grains in the 2005 dietary guidelines, played a critical role in achieving gains in this area. Data from other countries highlight the importance of a coherent national nutrition policy. Specifically, Finland implemented a national nutrition policy that included recommendations for the increased consumption of whole grains. A recent study observed that the implementation of these recommendations was associated with a significant increase in whole-grain consumption among adults [45]. Perhaps the largest success story related to increasing whole-grain intake is Denmark, where intake increased more than two-fold from 2007 to 2019 [46]. Highlighting the fact that dietary guidelines are just a first step, the incorporation of specific whole-grain recommendations in the Danish food-based dietary guidelines was complemented by a robust public–private partnership and clear food labeling [47].

While dietary guidelines appear promising as an avenue through which to increase whole-grain intake, the absence of a standardized definition for whole-grain foods poses challenges in promoting their consumption through consumer guidance, industry reformulations, and policy actions. The findings of this present study and those of other research indicate that a significant proportion of respondents struggle to differentiate between foods containing substantial amounts of whole grains and those with only minimal amounts (or even none) [48]. The lack of a consistent definition makes it difficult for the industry to reformulate products and increase the availability of whole-grain options in the market. Additionally, the inconsistency in defining whole-grain products creates obstacles for governments in formulating policies to promote whole-grain intake.

Inadequate and inconsistent food labeling is a further challenge. South Africa is not unique in not having officially formulated and published requirements or guidelines for labeling whole-grain products. This has resulted in varied practices related to the labeling of whole-grain products and may lead to confusion among consumers. For example, research from Australia suggests that consumers are skeptical of whole-grain information provided on the food product label by manufacturers and even of “healthy food product”-type logos from government agencies [10]. An alternative is to have legislated detailed labeling of whole-grain type and content.

In South Africa, “Whole grain” is defined by its Department of Health as “grains from cereals, which, after milling (if milled), naturally contain all the components, namely endosperm, bran, germ and all the macronutrients, micronutrients and trace elements of the original unprocessed whole kernel” [19]. Recent draft labeling regulation for whole grains in South Africa is unique in its approach as it distinguishes and defines three different types of whole grains, namely: whole grain, partially whole grain, and whole-grain flour/meal [20]. The draft labelling regulations state that if a food product contains recombined or whole-grain flour/meal, it can use the claim “wholegrain” but must specify “recombined” before it, followed by “flour” or “meal” depending on the case. The content claim must also include the percentage of whole-grain or partially whole-grain, as well as the Glycemic Index (GI) category. A logo for the whole-grain concept can only be used if the product consists of at least 97% whole grains. Alternatively, a logo is permitted if the final product contains a minimum of 75% whole grains. These definitions differ from the globally accepted definition of whole grains and introduce the concept of glycemic index, which should be considered separately. Such complex definitions and recommendations pose challenges in terms of educating consumers and lead to a lack of awareness.

A complementary approach is independent third-party certification of food products. A long-established example of this is the simple-to-understand Whole Grain Stamps of the Whole Grains Council [49]. There is some evidence that this latter approach can be useful in developing countries. In Latin America, the number of registered food products carrying the Whole Grains Stamp increased from approximately 100 when it was introduced in 2009 to more than 1300 in 2020 [49]. Further, a survey commissioned by the Whole Grains Council revealed that 2⁄3rds of consumers report that third-party labeling on foods gives them more confidence in the product, and more specifically that 86% of consumers trusted the Whole Grain Stamp [50].

Another issue, again not unique to South Africa, is that the number or amount of whole grains/whole-grain products to be consumed per day is not specified in the country’s Food Based Dietary Guidelines [51]. This means that consumers do not know how much whole grain they need to consume, and this is also not covered in the country’s draft whole-grain labeling regulations [20]. However, in view of the finding of this study that the respondents generally underestimated the quantity of whole grains that should be consumed, there is additionally a need for reliable information on the content of whole grains in a food product relative to recommended intake. There is emerging consensus that quantitative recommendations, such as those implemented in Denmark and the United States, are much more effective than qualitative recommendations (e.g., simply advising consumers to “eat more” whole grains) [42,47].

Economic incentives or disincentives may also help to nudge people towards increasing consumption of whole-grain foods. In South Africa, for example, only a few grain products, such as refined maize meal and wheat flour, are exempted from value-added tax (VAT), currently levied at 15%. The extension of sales tax exemptions to whole-grain products such as whole-grain maize, wheat and other local grains or more nutritionally dense products, such as whole-grain cereal–legume blends, would make these products more affordable and encourage less affluent consumers to include them in their diet.

A further major finding of this study was that the respondents currently know very little about the actual benefits of whole-grain food consumption. This strongly indicates that proactive strategies are additionally required. There is evidence that the provision of whole-grain foods in school nutrition schemes, in conjunction with social and behavior change communication (SBCC) initiatives, is a promising strategy in a low-consumer-income environment. In Rwanda, fortified whole-grain maize ugali (stiff porridge) is being provided in school nutrition schemes. This is being accompanied by in-depth SBCC, where the school children and their parents receive detailed information about whole grains and their nutritional and health-related benefits. A three-month study revealed that children aged approximately 10–13 y who received the SBCC information demonstrated a clear and significant preference towards fortified whole-grain maize (Darshana Joshi, Vanguard Economics, Rwanda, personal communication). Concerning the impact on their parents, although the parents had previously been informed about whole grains, there was evidence that SBCC further improved their overall knowledge. While in a very different context, these findings are like the US experience, emphasizing the important roles that schools play. A recent USDA Economic Research Service report on whole-grain intake in the US concluded that “Behavioral strategies such as taste tests, modeling by teachers and other role models, and promotion of social norms favoring healthy foods may further encourage [whole grain] acceptance” [44].

### 4.4. Strengths and Limitations of the Study

The major contribution of this work is that to date it is likely the largest and most systematic study of consumer knowledge and attitudes to whole grains and their foods. This is certainly the case in the context of consumers from culturally diverse middle-income countries. Further, it is the first study to provide data on consumers’ subjective (perceived) knowledge concerning whole grains versus their objective (actual) knowledge. The major limitation of the study is that only consumers of higher socioeconomic status were surveyed.

## 5. Conclusions

In order to effectively and sustainably increase the quantity of whole grains in the diets of people in developing countries like South Africa, collaborative efforts between the government, academia, and the industry are essential. The following steps are identified as promoting whole-grain consumption as a core component of a healthy diet:

- Consistent labeling: It is crucial to ensure that the labeling of whole-grain products aligns with the global approach, such as that followed by the Whole Grains Council. Standardizing the labeling requirements will provide clarity to consumers and enable them to make informed choices.
- Specification in dietary guidelines: To further support whole-grain consumption, it is recommended that the Food-Based Dietary Guidelines specify the recommended amounts of whole grains to be consumed per day. Clear guidelines will assist consumers to understanding the appropriate intake levels and incorporate whole grains into their diets.
- Increased consumer awareness: A government-led communication campaign should be implemented to enhance consumer awareness of the health benefits associated with whole grains. This campaign can educate the public about the nutritional value of whole grains and promote their inclusion in daily meals.
- Affordability through taxation exemption: To make whole-grain products more affordable for consumers, taxation exemptions should be extended to products containing significant amounts of whole grains. This would ensure that the prices of these products are comparable to those of regular items, encouraging consumers to choose whole-grain options.
- Research and development of appealing whole-grain products: Collaboration between the industry and academia is crucial for the development of good tasting and nutritionally dense whole-grain products. To meet consumer expectations, these products should be shelf-stable and less prone to rancid off-flavor development. Research efforts should focus on improving the taste and texture of whole-grain products to make them more appealing to a wider consumer base.

## Figures and Tables

**Table 1:** Participant characteristics.

	N	%
Total	1000	-
Age group (y)		
18–24	179	17.9
25–34	310	31.0
35–44	223	22.3
45–54	167	16.7
55–64	121	12.1
Gender		
Female	503	50.3
Male	497	49.7
Population group		
Black	764	76.4
Coloured/Indian	130	13.0
White	106	10.6
Living Standards Measure (LSM) ^1^		
5–7	597	59.7
8–10	403	40.3
Region (Province)		
Gauteng	383	38.3
KwaZulu-Natal	153	15.3
Western Cape	127	12.7
Other Regions	337	33.7
Self-reported health		
Excellent	185	18.5
Very good	287	28.7
Good	396	39.6
Fair/poor	132	13.2

^1^ LSM is a tool to provide a composite measure of socio-economic status in South Africa.

**Table 2:** Comparison of the subjective and objective knowledge of respondents as influenced by grouping characteristics.

Characteristics		n (% of Total)	% Respondents with Low or High Subjective Knowledge ^1^ of Whole Grains		Mean (±Standard Deviation [SD]) ^2^		
					Objective Knowledge about Items That Are Whole Grains?	Objective Knowledge about Products Containing Whole Grains?	Total Objective Knowledge Score ^3^
			Low	High	Max = 20	Max = 26	Max = 47
Subjective Knowledge ^1^	High	640 (64%)	-	-	10.5 (±2.1)	17.6 (±2.7)	28.5 (±4.0)
	Low	360 (36%)	-	-	10.1 (±2.0)	17.4 (±2.4)	27.8 (±3.6)
*p*-value			-	-	0.001	0.22	0.005
Gender	Female	503 (50.3%)	34.5	65.4	10.6 (±2.0)	18.1 (±2.6)	29.1 (±3.7)
	Male	497 (49.7%)	37.4	62.6	10.1 (±2.1)	16.9 (±2.5)	27.4 (±3.8)
*p*-value			0.351		<0.0001	<0.0001	<0.0001
Age	18–24 years	179 (17.9%)	30	70	10.1 (±1.8) ^a,b^	17.5 (±2.2) ^a,b,c^	28.0 (±3.1) ^a,b,c^
	25–34 years	310 (31.0%)	37.1	62.8	10.6 (±2.1) ^b^	18.0 (±2.5) ^c^	29.0 (±3.6) ^c^
	35–44 years	223 (22.3%)	32.7	67.3	10.5 (±2.0) ^b^	17.6 (±2.9) ^b,c^	28.5 (±4.0) ^b,c^
	45–54 years	167 (16.7%)	43	57	10.0 (±2.2) ^a^	17.3 (±2.6) ^a,b^	27.7 (±4.1) ^a,b^
	55–64 years	121 (12.1%)	43.1	56.9	10.2 (±2.4) ^a,b^	16.7 (±2.7) ^a^	27.2 (±4.6) ^a^
*p*-value			0.009		0.021	<0.0001	<0.0001
LSM	LSM 5–7	403 (40.3%)	40.5	59.5	10.1 (±2.0)	17.2 (±2.6)	27.7 (±3.8)
	LSM 8–10	597 (59.7%)	29.3	70.7	10.7 (±2.1)	18.0 (±2.5)	29.2 (±3.7)
*p*-value			<0.001		<0.0001	<0.0001	<0.0001
All respondents		1000			10.3 (±2.1)	17.5 (±2.6)	28.3 (±3.8)

^1^ Subjective knowledge—Respondents that considered themselves not very or less knowledgeable than others about whole grains (Low); versus those that considered themselves as knowing what whole grains are or having expert knowledge (High). ^2^ Comparison by Kruskal–Wallis test with superscripts (^a,b,c^) indicating categories not sharing a letter are significantly different from each other (*p* < 0.05); ^3^ Total objective knowledge score range from 0–47.

## Data Availability

The de-identified data is available on request.

## Supplementary Materials

The following supporting information can be downloaded at: https://www.mdpi.com/article/10.3390/nu15163522/s1, S1 Whole grains survey questions.
