# Supplementary material for: South African Consumers’ Knowledge, Opinions and Awareness of Whole Grains and Their Health Benefits: A Cross-Sectional Online Survey
Source: Nutrients. 2023 Aug 10;15(16):3522. doi: 10.3390/nu15163522 (PMC10457809; doi:10.3390/nu15163522)
Supplement: Supplementary file 1 [file nutrients-15-03522-s001.zip › nutrients-2508818-supplementary.pdf]

SCREENING

We are looking for certain groups of people, so first let's confirm if you qualify to take part in the survey

Q1. Do you currently live in South Africa?

SINGLE MENTION

Yes	1	CONTINUE
No	2	CLOSE

Q2. Where in South Africa do you currently live?

SINGLE MENTION

Eastern Cape	1
Free State	2
Gauteng	3
KwaZulu-Natal	4
Limpopo	5
Mpumalanga	6
North West	7
Northern Cape	8
Western Cape	9

Q3. Do you, or any of your close family or friends, work for any of these types of companies or professions?

READ LIST. MULTIPLE MENTIONS. RANDOMISE ORDER

Advertising Agency	1	CLOSE
Marketing/ Sales/ Promotions Company	2	
Market Research Company	3	
Public relations	4	
TV/ Radio/ Press/ Journalism	5	
Professions requiring a formal qualification in Food Science and Technology, Nutrition or Dietetics	6	
Company that makes/ distributes/ sells breakfast cereals/ porridges	7	
None of the above	8	CONTINUE

Q4. How old are you?

SINGLE MENTION

17 years or younger	1	CLOSE
18-24 years	2	CONTINUE
25-34 years	3	
35-44 years	4	
45-54 years	5	
55-64 years	6	
65 years or above	7	CLOSE

Q5. Are you…?

SINGLE MENTION

Female	1	CHECK QUOTA
Male	2	
Nonbinary/ Prefer not to answer	3	

Q6. How would you describe your population group?
SINGLE MENTION

Black	1	CHECK QUOTA
Mixed Race / Coloured	2	
Indian	3	
White	4	
Other (please specify)	5	

SHORT LSM6

These next few questions will allow us to better understand your lifestyle and are therefore only for classification purposes.

QLSM1: Do you own a personal computer such as a laptop or desktop?
SINGLE MENTION

Yes	1	CONTINUE
No	2	ASK QLSM4

QLSM2: Do you own a motor vehicle?
SINGLE MENTION

Yes	1	CONTINUE
No	2	ASK QLSM5

QLSM3: Do you have hot running water from a geyser in your home?
SINGLE MENTION

Yes	1	CLASSIFY LSM 8-10
No	2	CLASSIFY LSM 5-7

QLSM4: Do you own a TV?
SINGLE MENTION

Yes	1	CONTINUE
No	2	ASK QLSM6

QLSM5: Do you own a floor polisher or vacuum cleaner?
SINGLE MENTION

Yes	1	CLASSIFY LSM 8-10
No	2	CLASSIFY LSM 5-7

QLSM6: Do you have an electric stove or gas stove with an oven??
SINGLE MENTION

Yes	1	CLASSIFY LSM 5-7
No	2	CLASSIFY LSM 1-4

Q7. RECORD LSM

LSM 1-4	1	CLOSE
LSM 5-7	2	CHECK QUOTA
LSM 8-10	3	

WHOLE GRAINS

Q8. Which of these phrases best describes how well you understand the term "whole grains"?
SINGLE MENTION

Among my circle of friends, I am the expert on whole grains	1
I know what whole grains mean	2
I do not feel very knowledgeable about whole grains	3
When it comes to whole grains, I really do not know a lot.	4
Compared to most other people, I know less about whole grains	5

Q9. Which of the following best describes what you understand the term "whole grains" to be?
RANDOMISE ORDER. SINGLE MENTION

Grains which are genetically modified (created or modified through genetic engineering)	1
Grains which are grown organically/ with restricted use of pesticides and fertilizers	2
Grains which have undergone processing to add certain nutrients	3
Grains with all the original, edible parts present in the same proportion, as when the grain was growing in the fields	4
Any other grains apart from wheat and rice	5
A mixture of multiple grains (Multi-grains)	6
Flour made from intact grains	7
Ancient grains	8
Uncracked kernels	9
Other (please specify)	10
Don't know/ unsure	11

Q10. Which of the items listed below do you understand to be whole grains?
RANDOMISE ORDER. MULTIPLE MENTION

Barley	1
Brown rice	2
Buckwheat	3
Chia	4
Chickpeas	5
Cous-cous	6
Flax	7
Maize	8
Millets	9
Oats	10
Quinoa	11
Rye	12
Sorghum	13
Soybean	14
Spelt	15
Sunflower seed/ pumpkin seeds/ sesame seeds	16
Teff	17
Nuts (Cashew, Peanuts, Pecan, Macadamia, etc.)	18
Wheat	19
White rice	20

Other (specify)	21
None of the above	22

Q11. And which of these products do you believe generally contain whole grains?
RANDOMISE ORDER. MULTIPLE MENTION.

2-minute noodles	1
Almond milk	2
Breakfast cereals	3
Brown bread	4
Brown rice	5
Ciabatta/ French loaf	6
Corn flakes	7
Cous-cous	8
Granola bars	9
Low GI bread	10
Mabele/ Maltabella porridges	11
Maize meal	12
Pasta	13
Popcorn	14
Rolled oats/ oat flakes	15
Rye bread	16
Samp	17
Soybean milk	18
Oat porridge	19
Trail mix/ nuts & seeds	20
Weetbix	21
White bread	22
White wheat flour	23
White rice	24
Whole-wheat bread	25
Whole-wheat flour	26
Whole-wheat pasta	27
Other (Specify)	28
None of the above	29

Q12. Which of the following do you understand to be the benefits of consuming whole grains? Please select up to 5.
RANDOMISE ORDER. MULTIPLE MENTION. MUST SELECT AT LEAST ONE. SELECT UP TO 5 OPTIONS

Better gut/ bowel health	1
Better weight maintenance	2
Healthy skin and bones	3
Helps me lose weight	4
Keeps energy levels up	5
Prevents/ alleviates constipation	6
Lower risk of cancer	7
Makes you feel full for a longer period of time	8

Prevents/ treats numbness/ tingling in hands and feet	9
Reduced risk of heart disease	10
Reduced risk of stroke	11
Reduced risk of type 2 diabetes	12
Other (please specify)	13
None of the above/ Don't know	14

Q13. Which of the following attributes do you associate with whole-grain products? Please select up to 5 that you most associate with whole grains.
RANDOMISE ORDER. MULTIPLE MENTION. MUST SELECT AT LEAST ONE. SELECT UP TO 5 OPTIONS

Affordable	1
Bland	2
Boring	3
Brown	4
Chewy	5
Crunchy	6
Difficult to find where I shop	7
Difficult to identify from packaging	8
Dry	9
Easy to find where I shop	10
Easy to identify from packaging	11
Expensive	12
For older people	13
Healthy	14
Heart health	15
Low GI	16
Lowers my cholesterol	17
Poor taste	18
Quick and easy to prepare/ cook	19
Rough/ dry texture	20
Slimy	21
Tasty	22
Time-consuming to prepare/ cook	23
Other (please specify)	24
None of the above	25

Q14. Medical experts recommend eating whole grains for a balanced diet. Are you aware how much whole grains are recommended for daily consumption?
SINGLE MENTION

Equivalent to half a bowl of breakfast cereal	1
Equivalent to one bowl of breakfast cereal	2
Equivalent to one bowl of breakfast cereal plus one slice of bread	3
Equivalent to one bowl of breakfast cereal plus two slices of bread	4
Equivalent to one bowl of breakfast cereal plus four slices of bread	5
Don't know	6

Q15. In your opinion, are you consuming sufficient whole-grain foods?
SINGLE MENTION

Far too little	1
Too little	2
Almost enough	3
Enough	4
Plenty enough	5

Q16. Which of the following brands do you most associate with the term “whole grains”? Select up to 5 that you most associate with whole grains
RANDOMISE ORDER. MULTIPLE MENTION. RANK TOP 5
Select at least one

Bokomo	1
Jungle	2
Kellogg’s	3
Otees	4
Weetbix	5
Pronutro	6
Futurelife	7
White Star	8
Ace	9
Nutrific	10
Nestle	11
Golden Cloud	12
Sasko	13
Albany	14
Nature’s choice	15
Woolworths	16
Snowflake	17
Fatti’s and Moni’s	18
Koo	19
Blue Ribbon	20
King Korn	21
Monate	22
Tastic	23
Other (specify)	24
None of these	25

ATTITUDES TO HEALTH AND WELLNESS

Q17. In general, would you say your health is…?
SINGLE MENTION

Excellent	5
Very good	4
Good	3
Fair	2
Poor	1
